# Universal Applicator for Digitally-Controlled Pressing Force and Impact Velocity Insertion of Microneedles into Skin

**DOI:** 10.3390/pharmaceutics10040211

**Published:** 2018-11-01

**Authors:** Mara Leone, Bart H. van Oorschot, M. Reza Nejadnik, Andrea Bocchino, Matteo Rosato, Gideon Kersten, Conor O’Mahony, Joke Bouwstra, Koen van der Maaden

**Affiliations:** 1 Division of BioTherapeutics, Leiden Academic Centre for Drug Research, Leiden University, Einsteinweg 55, 2333 CC Leiden, The Netherlands; m.leone@lacdr.leidenuniv.nl (M.L.); Reza.Nejadnik@sanofi.com (M.R.N.); gideon.kersten@intravacc.nl (G.K.); bouwstra@lacdr.leidenuniv.nl (J.B.); 2 uPRAX Microsolutions B.V., Molengraaffsingel 12, 2623 JD Delft, The Netherlands; bart.van.oorschot@uprax.nl; 3 Tyndall National Institute, University College Cork, T12 R5CP Cork, Ireland; andrea.bocchino@tyndall.ie (A.B.); matteorosato00@gmail.com (M.R.); conor.omahony@tyndall.ie (C.O.); 4 Institute for Translational Vaccinology, Antonie van Leeuwenhoeklaan 9, 3721 MA Bilthoven, The Netherlands

**Keywords:** microneedle arrays, impact applicator, pressing force applicator, skin penetration, (trans)dermal drug/vaccine delivery

## Abstract

Microneedle technologies have been developed for dermal drug and vaccine delivery, including hollow-, solid-, coated-, and dissolving microneedles. Microneedles have been made in many different geometries and of many different materials, all of which may influence their skin-penetrating ability. To ensure reproducible and effective drug and vaccine delivery via microneedles, the optimal insertion parameters should be known. Therefore, a digitally-controlled microneedle applicator was developed to insert microneedles into the skin via impact insertion (velocity) or via pressing force insertion. Six microneedle arrays with different geometries and/or materials were applied onto ex vivo human skin with varying velocities or pressing forces. Penetration efficiency and delivered antigen dose into the skin after application of microneedles were determined. In general, microneedles pierced the skin more efficiently when applied by impact application as compared to application via pressing force. However, the angle of application of the applicator on the skin can affect the velocity of the impact, influencing the penetration efficiency of microneedles. Regarding the antigen delivery into the skin, the delivered dose was increasing by increasing the velocity or pressure, and thus, increasing the penetration efficiency. These data demonstrate that an applicator is an important tool to determine optimal application conditions with ex vivo human skin.

## 1. Introduction

Drug and vaccine delivery via the skin offers several advantages over conventional administration routes (i.e., oral and parenteral), including prevention of drug and vaccine degradation by the gastro-intestinal tract, elimination of pain and discomfort, acceptability by people with needle-phobia, avoidance of hazardous waste, needle-stick injuries, and needle re-use [1,2,3,4,5,6,7]. However, the top layer of the skin, the stratum corneum, limits delivery via the skin of high-molecular weight drugs and vaccines (>500 Da), as well as biotherapeutics, such as peptides, proteins, hormones, and growth factors [6,7,8]. To enable drug and vaccine delivery via the skin, different techniques, such as powder and fluid jet injection, thermal and liquid microporation, sonoporation, and microneedles have been proposed [9,10,11,12]. The use of microneedles has been shown to be very attractive to overcome the stratum corneum, thereby enabling drug and vaccine delivery into and through the skin. Microneedles are needle-like structures shorter than 1 mm. Depending on their material, sharpness, and method of application, microneedles can penetrate the skin, thereby creating transient pores reaching the epidermis or dermis but not reaching pain receptors, enabling pain-free intradermal delivery of macromolecules [2,5,13,14]. Different microneedle technologies have been developed for drug and vaccine delivery via the skin, including (a) hollow microneedles for injections of liquid drugs or vaccine formulations, and (b) solid microneedles classified as (i) microneedles for skin pretreatment, and (ii) porous, coated, hydrogel-forming microneedles and dissolving (polymeric) microneedles that, after insertion into the skin, release the drug or vaccine [15,16,17,18]. These different microneedle types have been made with many different geometries (e.g., length, sharpness, diameter, density), and have been made of different materials (e.g., glass, silicon, stainless steel, titanium, sugar, (synthetic) polymer), which all may influence their skin penetrating ability.

The two frequently used methods of microneedle application are by pressing force or impact application [6]. Application by pressing force can be performed manually [19,20,21,22] or by an applicator [23]. To apply microneedles by impact application, using a predetermined velocity or impact energy, an applicator is needed [24,25,26]. It has been shown that an impact applicator [26] or a controlled pressing force applicator [23] ensures improved reproducibility of microneedle piercing of the stratum corneum, independent of the user, compared to manual application by pressing force of the thumb.

The aims of this study were to (1) develop a microneedle applicator that supports control of the parameters to insert microneedles via impact application or pressing force into skin, and (2) evaluate the applicator for optimal microneedle insertion using a panel of different microneedle arrays (MNAs). Therefore, in this study a digitally-controlled microneedle applicator was developed, and was subsequently used to optimize the piercing of skin by different types of solid microneedles (i.e., different materials and geometries). The universal applicator uses an electronically-controlled unit to insert microneedles into the skin either by impact-insertion with a variable impact velocity, or by applying a variable pressing force. To evaluate the optimal insertion parameters for a variety of microneedle types, staining of the skin with trypan blue was performed to investigate the microneedle penetration efficiency, while an ovalbumin formulation was used to demonstrate efficacy of antigen delivery into the skin after microneedle application as pretreatment. This work shows that each microneedle type has different specific settings (impact velocity or pressing force) for optimal application on the skin.

## 2. Materials and Methods

### 2.1. Materials

Milli-Q water (18.2 MΩ/cm, Millipore Co., Bedford, MA, USA) was used for the preparation of aqueous solutions. Trypan blue solution 0.4% (*w*/*v*) and infrared dye (IRDye 800CW) were purchased from Sigma Aldrich and LI-COR (Lincoln, NE, USA), respectively. Polydimethylsiloxane (PDMS, Sylgard 184) was obtained from Dow Corning (Midland, MI, USA). 10 mM phosphate buffer (7.7 mM Na_2_HPO_4_, 2.3 mM NaH_2_PO_4_, pH 7.4) was prepared in the laboratory. Hyaluronan (sodium hyaluronate, average Mw was 150 kDa) was purchased from Lifecore Biomedical (Chaska, MN, USA). Vinylpolysiloxanes A-silicone (Elite Double 32a Normal) was purchased from the Zhermack Group (Badia Polesine, Italy) and two-component epoxy glue from Bison International B.V. (Goes, The Netherlands). 4 × 4 silicon microneedle arrays were obtained from Tyndall National Institute (Cork, Ireland), 24 × 24 silicon microneedle arrays were gifted from Bosch GmbH (Stuttgart, Germany), and ceramic alumina microneedle arrays (MLT-200 and MLT-475) were obtained from MyLife Technologies (MLT, Leiden BioScience Park, Leiden, The Netherlands). Conventional transparent tape Avery 440 Gloss Transparent Removable was obtained from Avery Dennison (Glendale, CA, USA).

### 2.2. Human Skin

Human abdomen skin was obtained within 24 h after cosmetic surgery from local hospitals after informed consent from the donors, and handled according to the Declaration of Helsinki Principles. After removal of the fat, the skin was stored at −80 °C until use. Prior to the microneedle application studies, the skin was thawed in an incubator at 37 °C for one hour in a petri dish with a wet tissue to prevent dehydration. Next, the skin was stretched and fixed on Parafilm-covered Styrofoam. Finally, the skin was sequentially cleaned with 70% ethanol and Milli-Q water.

### 2.3. Production of Dissolving Microneedle Arrays

**Figure 1:**
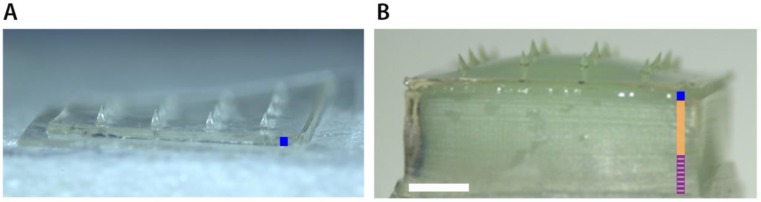
Bright field microscopy image (1.25× magnification) of a hyaluran dissolving microneedle array (dMNA) without (**A**) and with a bi-layered back-plate (**B**). Dark blue lines in (**A**,**B**) indicate the thin hyaluronan (HA) layer, the bright orange and purple stripped lines in B indicate silicone and glue layers, respectively. Scale bar (white line) represents 1 mm.

Dissolving MNAs (dMNAs) were fabricated by micromolding technique, pouring a solution of 10% (*w*/*v*) hyaluronan (HA) in 10 mM phosphate buffer (pH 7.4) in a PDMS mold. The PDMS mold was prepared by pouring and letting cure a PDMS solution consisting of a mixture of a silicone elastomer and silicone elastomer curing agent (10:1 ratio) on a template presenting Tyndall MNAs [27] (see Table 1). dMNA and dMNA featuring a back-plate part (dMNA-BP) of vinylpolysiloxane and epoxy glue were prepared using two different PDMS mold designs (Figure 1) [27].

### 2.4. Microneedle Arrays

In this study, a set of microneedle arrays with varying properties (e.g., microneedle length, microneedle geometry, density, surface area, material, etc.) were obtained or prepared (see Table 1) to evaluate their piercing ability using the newly-developed applicator (see sections below).

Microneedle were examined by scanning electron microscopy (SEM, Nova NanoSEM-200, FEI, Hillsboro, OR, USA) after coating with a layer of 15 nm platinum/palladium (Sputter Coater 208HR, Cressington, Watford, UK). The instrument was operated at 5.00 kV, and images were taken at magnifications of 80, 300, and 10,000 times. The tip diameters of microneedles were measured on SEM images using ZEN 2011 blue edition software, version: 2.0.14283.302 (Carl Zeiss Microscopy GmbH).

### 2.5. Applicator Design

**Figure 2:**
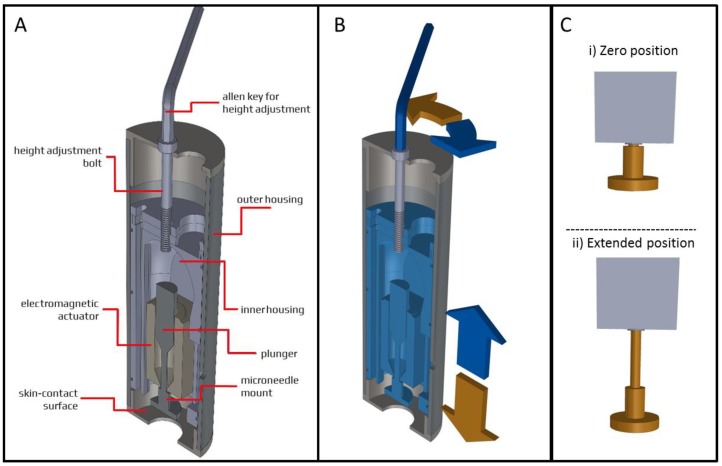
Design of the applicator (**A**) of which the inner housing (including the microneedle mount onto which microneedle arrays (MNAs) can be attached) can move towards the skin-contact surface by turning an allen key counterclockwise (brown arrows) and moving upwards by turning the allen key clockwise (blue arrows) (**B**). The electromagnetic actuator moving the microneedle mount (**C**).

A microneedle applicator was developed (uPRAX Microsolutions B.V., Delft, The Netherlands), as shown in Figure 2, to apply microneedles either via pressing force or impact insertion into the skin (see sections below). As shown in Figure 2A, the applicator contains an electromagnetic actuator (solenoid). The mass *m_app_* of the movable part of the applicator (25 g) is important for calculating the impact energy (Equation (1)) upon microneedle impact, where *v* is the velocity of the microneedle mount at impact:

Impact energy = ½ × *m_app_ v*^2^ = *J* (1)

The movable part of the actuator contains a plunger (23 g) onto which a microneedle mount (2 g) is attached, which will move 1.3 cm (=stroke) in the vertical direction upon activation. The outer housing contains a skin-contact surface and a height-adjustment bolt that is connected to the inner housing. The electromagnetic actuator is mounted in the inner housing that has an adjustable position relative to the outer housing. The position of the inner housing is moved towards the skin contact surface by turning the allen key for height adjustment counterclockwise, and moving the inner housing upwards by turning the allen key clockwise (Figure 2B). The electromagnetic actuator has two important positions: (i) the zero position, when the actuator does not have (enough) electric power, and (ii) a maximum extended position, when the actuator has enough electric power to stay in this position (Figure 2C).

### 2.6. Pressing Force and Impact Insertion Application

**Figure 3:**
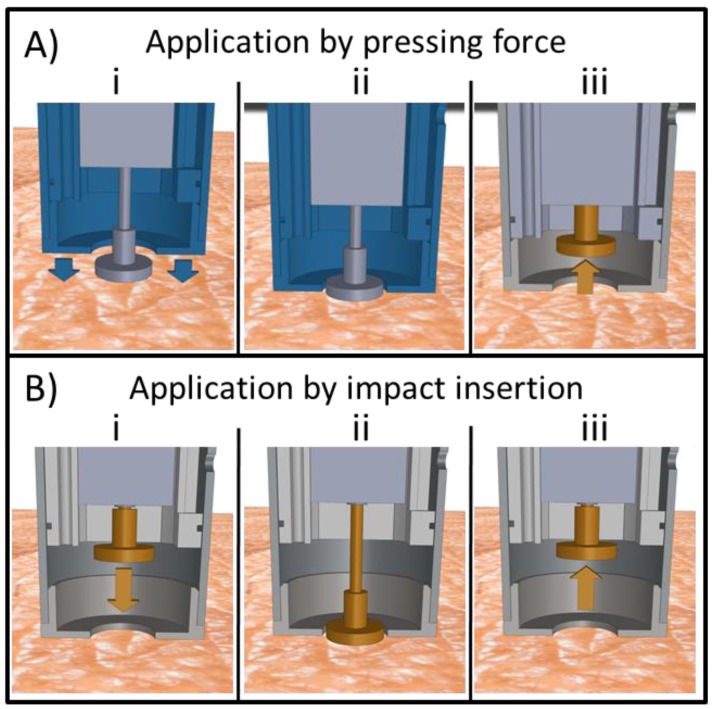
Application of microneedles onto skin by (**A**) pressing force: (**i**) electromagnetic actuator in the extended position, (**ii**) microneedle mount pressed onto the skin by hand force, (**iii**) microneedle mount retracted into the applicator; (**B**) impact insertion: (**i**) electromagnetic actuator in its retracted position, (**ii**) microneedle mount propelled into extended position, (**iii**) microneedle mount retracted into the applicator (see main text for explanation).

To apply MNAs by pressing force, the microneedle mount (onto which MNAs are attached (not shown in the image)) is protruding through the skin contact surface, while the electromagnetic actuator is in the extended position (Figure 3A-i). Next, the microneedle mount is pressed onto the skin by hand force (Figure 3A-ii). By exceeding the predetermined actuator’s holding force (see below), the microneedle mount is retracted into the applicator (Figure 3A-iii).

To apply MNAs by impact application, the position of the inner housing should be adjusted in such a way that the skin contact surface is in line with the microneedle mount when the electromagnetic actuator is in its maximum position (Figure 2C-ii). The applicator’s skin contact surface is first placed onto the skin when the electromagnetic actuator is in its retracted position (Figure 3B-i). Next, upon activation of the electromagnetic actuator, the microneedle mount moves towards the skin into the extended position with a predetermined impact velocity (see below) (Figure 3B-ii). Finally, the microneedle mount automatically retracts into the applicator upon reaching the set application time (Figure 3B-iii).

### 2.7. Microneedle Applicator Controller

A digitally-controlled applicator controller unit was developed to have precise control over the settings of microneedle application via the applicator. The applicator controller unit was programmed to modulate the duty cycle of a pulse width modulated (PWM) signal at a frequency of 10.5 kHz. The PWM signal was used to regulate the power of the applicator’s electromagnetic actuator by switching a 24 V, 1 A power source via a transistor. Based on the mode of application, pressing force, or impact insertion, the pulse width was modulated to adjust the actuators holding force (see below) or the movement of the microneedle mount from the zero position to the extended position (see Figure 2C), thereby regulating the impact velocity (see below for further explanation). Furthermore, when the applicator is used for impact application, the applicator controller unit provides control over the application time (i.e., the time that a microneedle is retained onto the skin).

### 2.8. Force Calibration for Pressing Force Application

To control the pressing force by which MNAs can be applied onto the skin, the holding force of the electromagnetic actuator was regulated. When the electromagnetic actuator is in its extended position (Figure 2C-ii), it has a holding force that is dependent on the electric power it receives. This means that the electromagnetic actuator will stay in the extended position until a pressing force applied onto the microneedle mount exceeds the solenoid’s holding force (the plunger will not be in its optimal electromagnetic field, and thereby, the force is reduced), which results in the actuator returning to the zero position (see Figure 2C-ii). To this end, the pressing force (*F_pressing_*_*force*_) at which the electromagnetic actuator retracts to the zero position was determined by using the Equation (2):

*F* = *m* × *g* (2)

where *m* is the mass measured by pressing the microneedle mount on a balance as a function of the pulse width value until microneedle mount detraction, and *g* is the gravitational acceleration.

The pressing force was fitted in GraphPad Prism 7 (GraphPad, San Diego, CA, USA) as a function of the duty cycle of the PWM using linear regression (*F_pressing force_ = slope × PWM − Force*). By programming these linear regression parameters (*A*, *B*) in the applicator controller unit, the pulse width was set for a chosen pressing force of the applicator.

### 2.9. Calibration of Impact Velocity for Impact Insertion

**Figure 4:**
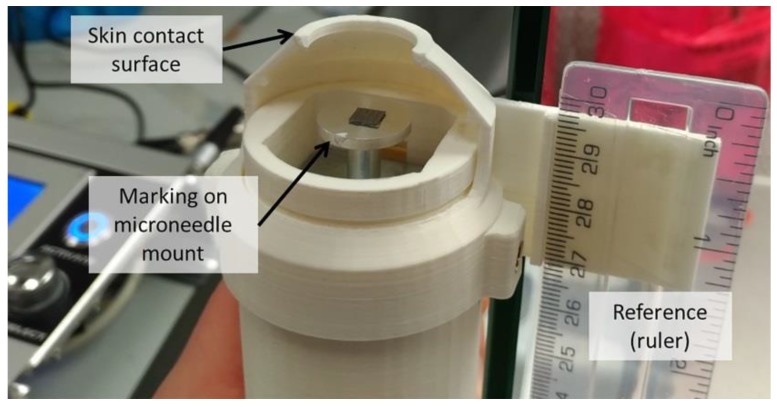
Microneedle applicator used for high-speed imaging.

In order to control the velocity at the impact of microneedle application, the average velocity as a function of the duty cycle of the PWM was determined. To this end, high speed imaging was used (FASTCAM Mini UX100, Motion Engineering Company, Inc. (Westfield, India) at 8000 frames per second). As shown in Figure 4, a reference (ruler) was attached to a 3D printed skin contact surface, and the microneedle mount was marked with a white spot. The position of this spot was tracked over consecutive frames to determine the traveled distance and the velocity of the microneedle mount as a function of time using the Tracker software version 5.0.1 (freely available from https:physlets.org/tracker). To calibrate the impact velocity of the microneedle applicator, high-speed imaging was performed in tenfold for each different pulse width (*PWM_width_*). The impact velocity (*v_imp_*) was defined as the average velocity at impact (which was at a displacement of the microneedle mount of 12.5–13 mm). Next, the *PWM_width_* was plotted as a function of *v_imp_*, which was fitted using nonlinear regression (*v_imp_ = (Y0 − Plateau) × exp(−K × PWM_width_) + Plateau*) in GraphPad Prism 7. The calibration parameters (*Y0*; *Plateau*; *K*) were programmed in the applicator controller unit to set the pulse width for a chosen impact velocity.

### 2.10. Influence of the Applicator’s Angle as a Function of the Pulse Width

The angle of an impact applicator related to the gravitational force might influence the impact velocity and impact energy of the applicator, and thereby, the penetration efficiency of MNAs. Therefore, the velocity of the electromagnetic actuator as a function of the pulse width was investigated under different angles ranging from 0°–180° (where at 0° the microneedle mount is moving in the same direction as the gravitational force and at 180° in the opposite direction). The impact velocity was determined using high-speed imaging and the tracking software as described above.

### 2.11. Application of MNAs onto Ex Vivo Human Skin

In this study, all microneedles were mounted onto the microneedle mount of the applicator by using double-sided adhesive tape (Tesa^®^). To investigate the optimal manner of application of a set of MNAs with different properties via impact insertion or pressing force, MNAs were applied with different velocities and pressing forces, respectively. MNAs applied by impact application were applied with 6 different impact velocities measured by high speed camera: 27, 42, 70, 97, 125, and 138 cm/s, and a fixed array retraction after 1.00 s. Each microneedle array was applied three times on a different site of ex vivo human skin from a single donor. The insertion parameters of different MNAs by pressing force was investigated by applying each array in triplicate using 6 different pressing forces: 1, 2, 5, 10, 17, and 25 Newton, and an application time of 5 s per microneedle.

### 2.12. Determination of Penetration Efficiency by a Trypan Blue Assay

To determine the penetration efficiency by the different microneedle arrays, a drop of 70 µL 1.2 mg/mL trypan blue was applied for 1 h on microneedle-pierced skin. Next, the stratum corneum was removed by tape-stripping until the skin appeared shiny (approximately 10 times stripping) to prevent overestimation of the penetration efficiency [26]. Subsequently, the skin was imaged by microscopy and the resulting microscopic images were analyzed using ImageJ software version 1.48v (freely available at http://rsb.info.nih.gov/ij/) to determine the amount of skin piercings. The penetration efficiency (*PE*) was calculated by using Equation (3) [26]:

*PE* = (number of piercings/number of microneedles per array) × 100% (3)

### 2.13. Calculation of Penetration Parameters

The MNAs used in this study had different properties. To calculate the optimal insertion parameters for the different microneedle geometries, the EC50 value for the different microneedle arrays was calculated per MNA, per individual microneedle, and MNA (back-plate) surface area. The EC50 value corresponded to the required impact energy or pressing force to pierce 50% of the microneedles of a MNA into the skin, and was calculated in GraphPad Prism 7 using a dose-response 3-parameter fit, which was constrained with: bottom asymptote penetration efficiency = 0; top asymptote penetration efficiency < 101 and hill slope < 0.2.

### 2.14. Normalization of Penetration Efficiency

To optimize the rate of antigen delivery and/or sampling when using MNAs, the MNA geometry may need adjustment (i.e., increase the number of microneedles, the surface area of an array or the microneedle length). This will lead to altered MNA insertion parameters. Therefore, to estimate the insertion parameters of a MNA when the number of microneedles or MNA surface area changes (while retaining a microneedle-specific geometry), we attempted to normalize the impact energy and pressing force per microneedle, with the assumptions that all microneedles from a single array pierce the skin in the same manner and the energy and force are homogeneously distributed per single microneedle of one array. Furthermore, when normalizing for the MNA (back-plate) surface area, we assumed that the energy and force are homogeneously distributed over the back-plate surface area (rather than on the individual microneedles).

### 2.15. Delivery of Fluorescently Labeled Ovalbumin into Pierced Skin

To investigate the relationship between the penetration efficiency and the delivery of an antigen into the skin as a function of the impact velocity and insertion force, the model antigen ovalbumin was used. To this end, ovalbumin was labeled with IRDye 800CW (OVA-IR800) according to the manufacturer′s instructions. A molar ratio of ovalbumin, IR800 of 2:1 (10 mg ovalbumin: 0.5 mg IRDye 800CW), was used. Next, 10 mg/mL ovalbumin in carbonate buffer (100 mM, pH 9) was added to 0.5 mg IRDye 800CW and pipetted in an Eppendorf Thermomixer R (Sigma-Aldrich, St. Louis, MO, USA) for 1 h at 300 RPM at room temperature. The unreacted dye was removed by using a 5 mL desalt column (LI-COR Biosciences, Lincoln, NE, USA). The infrared signal of labeled ovalbumin was measured on a Tecan Infinite M1000 plate reader (Männedorf, Switserland) at excitation and emission wavelengths of 774 nm and 789 nm, respectively. A concentration of 6.8 mg/mL OVA-IR800 (68% labeling efficiency) was obtained.

After application of the different MNAs with different impact velocity and application forces, a drop of 70 µL 20 mg/mL OVA-IR800 was applied. After 2 h, the excess of liquid was removed and the skin was tape-stripped. In order to quantify the dose of OVA-IR800 delivered into the skin after MNA pretreatment, a calibration curve of OVA-IR800 in ex vivo skin was prepared. Intradermal microinjections at a depth of 150 µm of an OVA-IR800 solution of 10 µg/mL with injection volumes ranging from 0.5–20 µL (5–200 ng) were performed, using a microinjection system as reported previously [28].

The near-infrared fluorescence of the delivered OVA-IR800 was measured in a Perkin-Elmer IVIS Lumina Series III in vivo imaging system (Waltham, MA, USA) using an indocyanine green background (ICG bkg) excitation filter and an ICG emission filter and an acquisition time 10 s. Living Image software version 4.3.1.0 (Perkin-Elmer, Waltham, MA, USA) was used for image acquisition and analysis. Fluorescence data were processed using region of interest (ROI) analysis, with background subtraction consisting of a control region of ex vivo human skin.

## 3. Results

### 3.1. Microneedles Appearance

**Figure 5:**
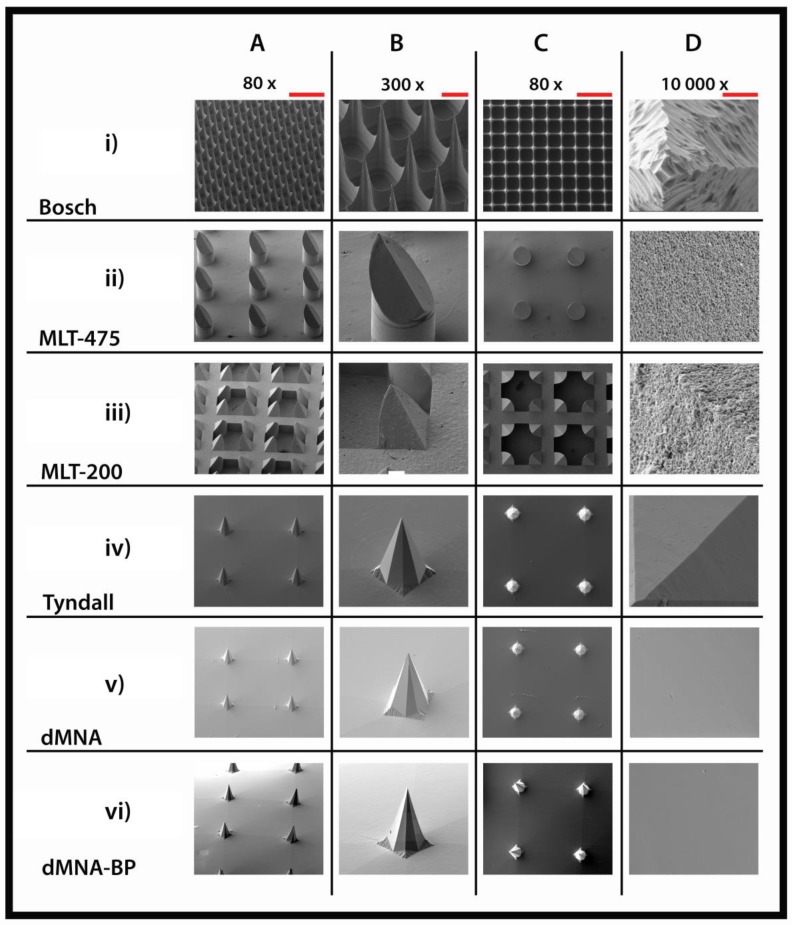
Scanning electron microscopy images of microneedle arrays (MNAs) with different geometries and lengths: Bosch of 320 µm (**i**), MLT-475 of 475 µm (**ii**), MLT-200 of 200 µm (**iii**), and Tyndall (**iv**) dMNA (**v**) and dMNA-BP (**vi**) of 300 µm. MNAs were imaged from a lateral view (**A**,**B**), and from the top side (**C**,**D**). Scale bars represent 500 µm (80×) (**A**,**C**), 100 µm (300×) (**B**), and 4 µm (10,000×) (**D**).

To visualize the differences in microneedle geometry and surface properties of the different MNAs, SEM imaging was performed (Figure 5), and the tip diameter was measured (Table 2). The Bosch MNAs have a high microneedle density, and the silicon surface is rougher than for other microneedle types. A Bosch tetrahedral structure presents a narrow tip diameter, making it the sharpest microneedle in the MNA set. MLT-475 and MLT-200 are nanoporous (made of alumina nanoparticles), and therefore, have a rough surface. The MLT-475 shape is similar to conventional hypodermic needles, whereas MLT-200 consists of groups of four needles positioned in a square. These two microneedle types present the largest tip diameter in comparison with the other microneedle types. Tyndall, dMNAs, and dMNAs-BP have a smooth surface and an identical octahedral geometry due to the PDMS mold that is based on the Tyndall MNAs to fabricate dMNAs and dMNAs-BP.

### 3.2. Applicator Setting: Velocity

**Figure 6:**
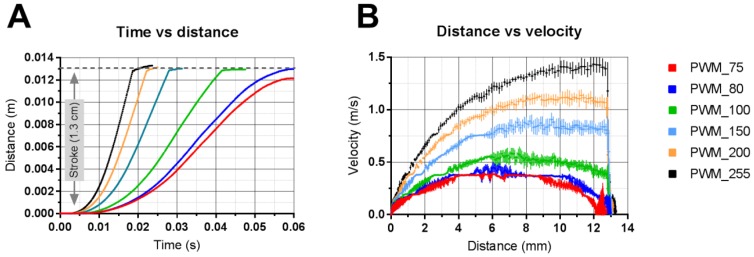
Analysis of high-speed imaging data (8000 fps) to determine the traveled distance (**A**) and the velocity as a function of the position of the microneedle mount at different pulse widths (**B**). Data are shown as mean ± SD, *n* = 10.

The relationship between applicator setting (pulse width) and output (velocity and travel distance) was determined by high-speed imaging in combination with tracking software. As shown in Figure 6A, the distance of the microneedle mount from the zero position (*S* = 0 mm) to the extended position (*S* = 13 mm) as a function of time was determined using different pulse widths. This shows that a pulse width of 80 (dimensionless 8 bit (0 to 255) value) or more is required to move the microneedle mount into the extended position, which is required to impact the microneedles onto the skin. Furthermore, from this figure, the time of flight (i.e., the time required to move the solenoid from the zero position to the extended position of 13 mm) was determined as a function of the pulse width. In Figure 6B, the traveled distance of the microneedle mount was plotted as a function of the velocity, showing that the maximum velocity of the microneedle mount was reached when the microneedle mount traveled a distance of 8–10 mm when using pulse widths of 150 or more. However, when using pulse widths of 100 or lower, the maximum velocity was higher than the impact velocity (because the spring in the solenoid, which returns the microneedle mount back in the zero position when there is no power on the solenoid, decelerates the microneedle mount). Therefore, to calibrate the applicator and to calculate the impact energy (see Table 3), the impact velocity was used.

### 3.3. Calibration of Applicator

**Figure 7:**
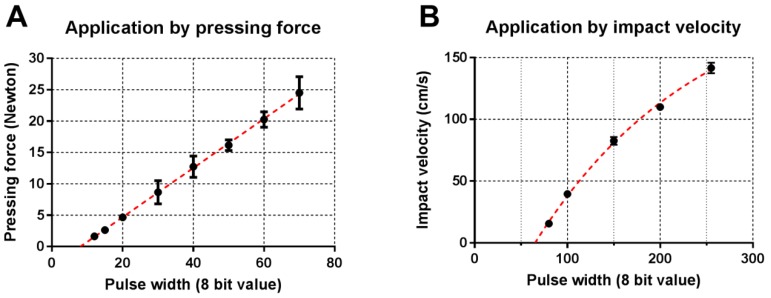
Regression curves used to set the applicator’s pulse width as a function of the chosen pressing force (**A**) and impact velocity (**B**) at a pulse frequency of 10.5 kHz. Data are shown as mean ± SD, *n* = 3 (**A**) and *n* = 10 (**B**).

The applicator was calibrated for pressing force and impact velocity to automatically set the pulse width for a chosen pressing force or impact velocity. To this end, the pressing force of the applicator was determined as a function of the pulse width, as shown in Figure 7A. Subsequently, the regression curve variables (*Force* = 0.392 × *PWM_width_* + 3.16) were programmed into the applicator controller unit to set the pulse width for a chosen pressing force between 1 and 25 Newton. Next, the applicator’s impact velocity (extracted from Figure 6) was plotted as a function of the pulse width, as shown in Figure 7B. To set the applicator’s velocity, the regression curve variables (*v_imp_* = (−0.9421 − 2.139) × exp(−0.005599 × *PWM_width_*) + 2.139) were programmed into the applicator controller.

In order to determine the reproducibility in impact velocity or pressing force delivered by the applicator, the relative standard deviation (RSD) per each impact velocity or pressing force value was calculated. Then, the set of RSD was averaged. An average RSD of 8.9% and 3.4% were obtained for pressing force and impact velocity application, respectively.

### 3.4. Influence of the Applicator’s Angle on the Impact Velocity

**Figure 8:**
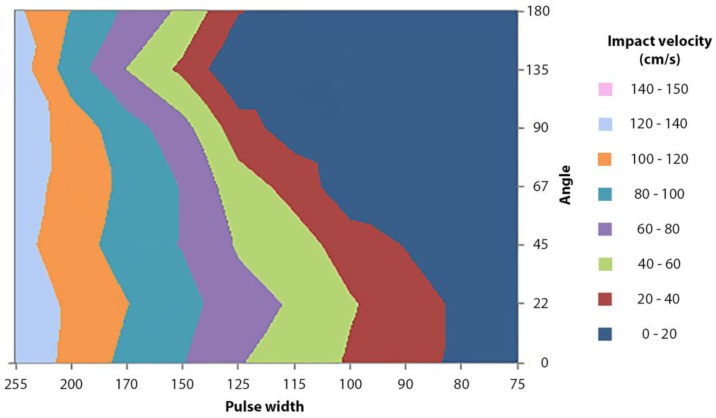
The impact velocity of the microneedle mount (in impact insertion mode) as a function of pulse width (8 bit dimensionless value; 0–255) and angle from the direction of the gravitational force (mean, *n* = 3, except for 0° which was *n* = 10).

The angle of an impact applicator relative to the gravitational force might influence the applicator’s impact velocity and impact energy, and thereby, the penetration efficiency of MNAs. Therefore, the applicator’s impact velocity was investigated as a function of the angle (related to the gravitational force) and pulse width (Figure 8). At low pulse widths (<80), the microneedle mount did not reach the extended position (Figure 6A); thus, no relation between impact velocity and applicator’s angle can be defined. At medium pulse widths (90–170), the impact velocity is highly dependent on the applicator’s angle. Finally, using high pulse widths (>170) results in reproducible velocities that are less dependent on the angle of the applicator.

### 3.5. Penetration of Human Skin by Pressing Force and Impact Application

**Figure 9:**
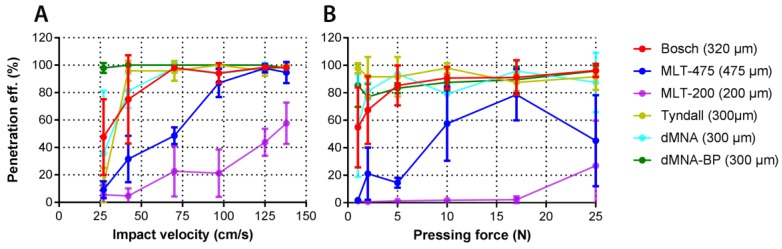
Penetration efficiency (in percentage) of MNs on ex vivo human skin as a function of the microneedle impact velocity (**A**) and pressing force (**B**). Data are shown as mean ± SD, *n* = 3.

The penetration of ex vivo human skin as a function of pressing force and impact application was investigated for six MNA designs (see Table 1 and Figure 9). The parameters that were used to apply the six different (MNAs) by impact application are summarized in Table 3.

For microneedles with a tetrahedral structure, like Bosch, and octahedral structure, like Tyndall, dissolving and dissolving-BP, both impact velocity and pressing force allowed a penetration efficiency of ex vivo human skin close to 100%. The structure and MNA surface area of MLT-475 allows a high penetration efficiency at an impact velocity above 100 cm/s. Nevertheless, application by pressing force resulted in a lower and less reproducible penetration efficiency. Similarly for MLT-200, a better penetration was obtained using the impact application as compared to pressing the MNAs into the skin. However, the design and high MNA surface area of MLT-200 did not result in higher penetration efficiencies than 60% using the settings wherein the applicator was calibrated.

Furthermore, the reproducibility in piercing at the best impact velocity or pressing force value per each MNA was investigated by calculating the RSD of the penetration efficiency at the highest impact velocity and pressing force values (Table 4).

### 3.6. Calculation of Penetration Parameters

Differences in MNAs geometry (microneedle shape and length, square or circular MNA, MNA area, and number and density of microneedles) and material (silicon, alumina, or hyaluronate) did not make possible a direct comparison of the penetration efficiencies of the different types of microneedles. However, the applicator could be set to find the optimal penetration properties to pierce the microneedles effectively and reproducibly (relatively low SD), and determine the piercing properties (EC50).

**Figure 10:**
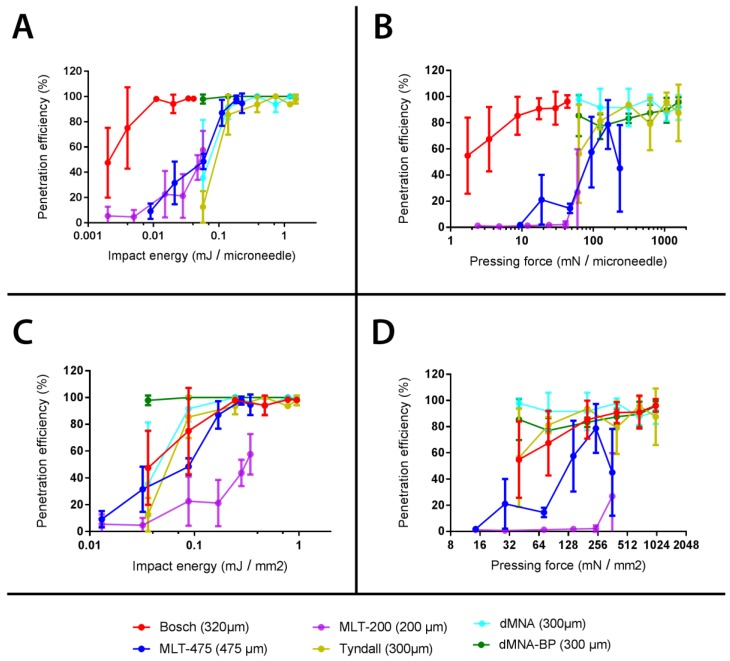
Penetration efficiency (in percentage) on ex vivo human skin as a function of the microneedle impact energy (**A**,**C**) and pressing force (**B**,**D**) both normalized for microneedle number (MN) (**A**,**B**) and microneedle array surface area (MNA surface) (**C**,**D**). Data are shown as mean ± SD (*n* = 3).

Penetration properties were calculated as EC50 values, and the corresponding pressing forces and impact energies were normalized per single microneedle and per MNA surface area (see Figure 10 and Table 5). A more reproducible piercing can be obtained when the applicator is set for impact velocity as compared to pressing force (Figure 10). When the application is in pressing force mode, the piercing is still effective, especially for high pressing forces and tetrahedral and octahedral geometries, but with reduced reproducibility.

### 3.7. Relation between Penetration Efficiency and Antigen Dose Delivered into the Skin

**Figure 11:**
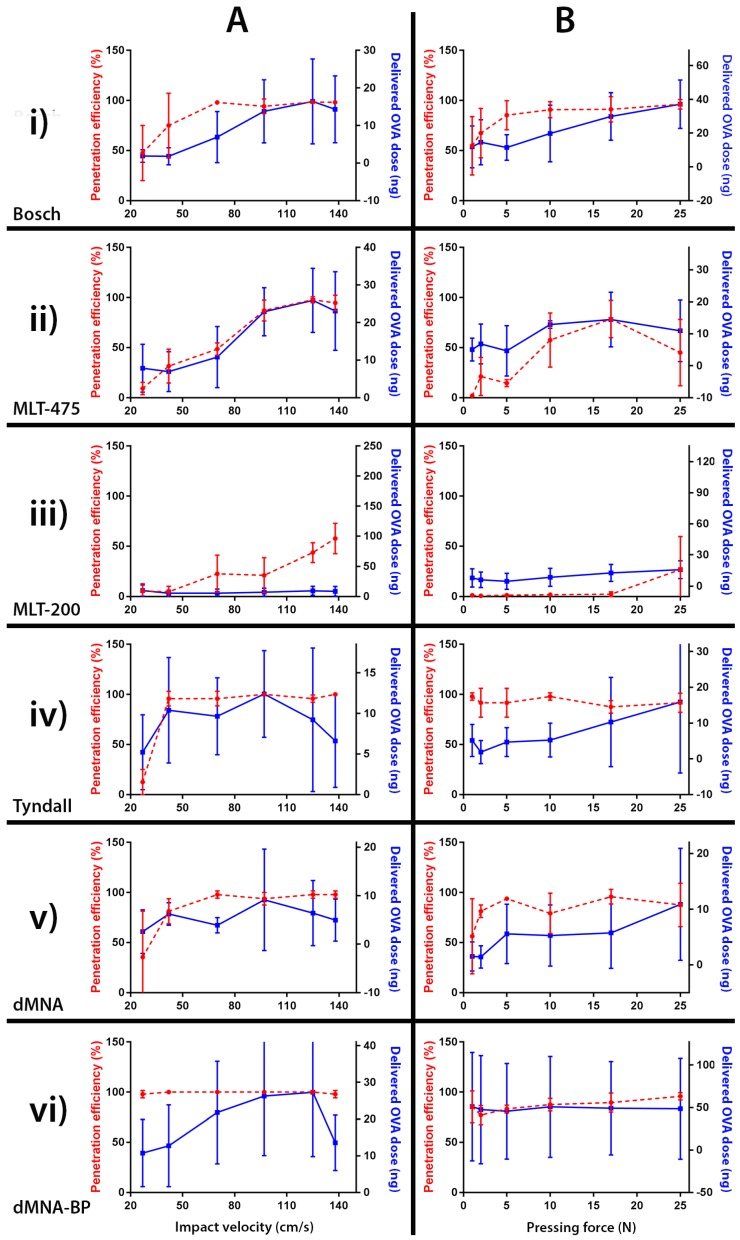
Penetration efficiency (in percentage) (red dotted line) and delivered dose of ovalbumin-IRDye 800CW (maximum dose delivery value matching the corresponding penetration efficiency value) (blue continuous line) into in vitro human skin as a function of the microneedle impact velocity (**A**) or pressing force (**B**). Six different microneedle arrays (MNAs) were used, having different geometries and lengths: Bosch of 320 µm (**i**), MLT-475 of 475 µm (**ii**), MLT-200 of 200 µm (**iii**), and Tyndall (**iv**) dMNA (**v**) and dMNA-BP (**vi**) of 300 µm. Data are shown as mean ± SD (*n* = 3). OVA: ovalbumin.

The relation between the penetration efficiency and the delivered dose of antigen into the skin as a function of the impact velocity and pressing force for different types of MNAs was investigated (Figure 11). For some MNAs, the penetration efficiency reached its maximum, but the amount of delivered ovalbumin was still increasing with further increasing impact velocities or application forces. Furthermore, despite fact that the penetration efficiency increased as a function of the impact velocity and the application force using the MLT-200 MNAs, the delivered amount of ovalbumin did not proportionally increase with the penetration efficiency. However, in general, the amount of ovalbumin delivered into the skin increased proportionally with an increasing penetration efficiency as a result of increasing impact velocities and pressing forces.

## 4. Discussions

In the literature, most microneedle applicators are used to apply MNAs via pressing force or impact application [6,19,20,21,22,23,24,25,26]. However, these applicators do not provide precise control over the pressing force or impact velocity of microneedle application. Applicators with precise control over such parameters can be used to get fundamental insights into critical application parameters of specific microneedle designs. Therefore, the aims of this study were to (1) develop a digitally-controlled microneedle applicator that supports control of the insertion parameters of microneedles into skin via impact application or via pressing force, and (2) evaluate the applicator for the optimal application of microneedles (effective and reproducible skin piercing) using a set of six different MNAs with varying properties (e.g., length, density, material, MNA surface).

### 4.1. Microneedle Applicator

It has been reported that long microneedles (>550 µm) could pierce skin effectively upon manual application, while microneedles of 300 μm and shorter generally require an impact applicator to pierce the skin effectively [29,30]. Importantly, the use of an applicator can result in lower inter- and intra-individual (several applications from the same person or from different people) variation as compared to manual application without an applicator [23,26]. Considering the reported studies, this work aimed to underline the importance of a device to assist the microneedle insertion if relatively short microneedles are used, and to determine the optimal settings (impact velocity and/or pressing force) for an efficient and reproducible piercing of different MNAs into the skin.

In order to achieve efficient and reproducible skin penetration, the delivered impact velocity and pressing force should be reproducible. In this study, it was shown that a chosen pressing force was reproducibly delivered by the applicator (average relative standard deviation of 8.9%), and was linearly dependent on the pulse width (Figure 7A). Furthermore, regarding impact application, it was shown that a pulse width below 80 did not allow the microneedle mount to reach the extended position, and will thereby not result in the microneedles impacting the skin. After accelerating of the microneedle mount (until approximately 4–8 mm) at pulse widths of 100 and lower, the velocity was decreasing towards the extended position (Figure 6B). However, using pulse widths between 90 and 255 resulted in very reproducible impact velocities (average relative standard deviation of 3.4%) between 30 and 140 cm/s (Figure 7B).

Skin penetration by microneedles is dependent on the impact velocity. However, in this study, it was shown that the angle of the applicator related to the gravitational force influences the impact velocity. Therefore, these data show the importance of designing applicators that deliver sufficient energy to reach an impact velocity that results in efficient and reproducible microneedle piercing, or ensure that impact applicators are only used at a specific angle.

Summarizing, the developed applicator was able to accurately deliver a chosen impact velocity and pressing force with high reproducibility.

### 4.2. Skin Penetration by Microneedles

For microneedle-based drug and vaccine delivery, it is important that microneedles pierce the skin effectively and reproducibly. In general, the investigated MNAs gave a high penetration efficiency both when applied by impact velocity and by pressing force. By using impact application, the penetration efficiency was mostly reaching 100%, while it reached 80% when using application by pressing force. This was also observed in our previous study using microneedles with a length of 200 µm (576 microneedles/array on a back-plate of 5 × 5 mm) [26]. This was very evident for MLT-200 and MLT-475: the impact application was more efficient compared to application by pressing force. This better performance in penetration efficiency by impact application may be related to the lower sharpness and to the roughness of the material (see SEM imaging) increasing the friction of the microneedle during penetration, or to the microneedle design, which has been shown to play an important role in mechanical properties of microneedles [20,31]. Furthermore, the microneedle length can have a strong impact on the penetration. In a previous study [26] Bosch microneedles with similar design as those used in the present study (MNA surface, density and material) showed an EC50 of 3.5 N/array with a lower penetration efficiency than the Bosch microneedles in the present study (EC50 1.4 N/array), which were only slightly longer, i.e., 320 µm vs 200 µm. Similarly, the MLT-200 µm microneedles require more than 25 N/array, whereas the longer MLT-475 µm needles had an EC50 of 8.0 N/array. Furthermore, applying microneedles by pressing force (17 and 25 N) (with respect to the penetration efficiency) resulted in reproducible skin penetration for Bosch, Tyndall, and dMNA(-BP) microneedles (Figure 9B). Moreover, using impact velocity (125 and 138 cm/s) resulted in very reproducible skin penetration for all investigated microneedles except MLT-200 (Figure 9A). Summarizing, applying microneedles via impact application generally results in higher penetration efficiencies with higher reproducibility.

It is interesting to note how the presence of a curved MNA surface (back-plate) can improve the penetration efficiency both by impact and pressing force application. This was the case of dMNA-BP differentiating from the dMNA only for the presence of a convex surface area where the MNs are located [27]. The convex surface may enable a better positioning of the microneedles towards the skin surface when pressed by the array, increasing the capability of microneedles to penetrate the skin.

The EC50 values calculated for the different microneedle geometries could be used as guidance for developing applicators for microneedles with altered numbers of microneedles and surface areas while retaining the microneedle geometry.

### 4.3. Delivery of a Model Antigen in Relation with the Penetration Efficiency

The standard deviations of the quantification of the delivered amount of antigen were rather high, which was partially caused by the measurement method (in this study the antigen-signal to background ratio was [1.3:1]) and by a short antigen application time on microneedle-treated skin, thereby not leading to a steady state diffusion. However, the amount of ovalbumin delivered in the skin showed that there is generally a relationship between the increase in penetration efficiency and increase in delivered amount of antigen with increasing impact velocities and pressing forces. Therefore, these data indicate that using the penetration efficiency could be a good value to estimate the required impact velocity and pressing force to obtain effective drug and vaccine delivery when using the “poke and patch” approach [32].

## 5. Conclusions

We have developed an applicator that reproducibly delivers a chosen pressing force and impact velocity. This applicator can be used to determine the optimal insertion parameters of a variety of microneedle arrays, and could therefore be a valuable tool for microneedle researchers. Moreover, using this applicator results in effective and reproducible piercing of ex vivo human skin with microneedles prepared with different shapes and materials.

## Figures and Tables

**Table 1:** Material and geometry of the microneedle arrays used in this study. Abbreviations: dissolving microneedle (dMNA); bi-layered back-plate (BP); microneedles (MNs).

Microneedle Array	Material	Array Geometry	Microneedle Length (µm)	Microneedle Density (cm^−1^)	Backplate Surface (mm^2^)	Number of MNs
Bosch	Silicon	Square (5 × 5 mm)	320	2304	25	576
MLT-475	Ceramic	Circular (d = 9 mm)	475	150	69	105
MLT-200	Ceramic	Circular (d = 9 mm)	200	600	69	414
Tyndall	Silicon	Square (5 × 5 mm)	300	64	25	16
dMNA	Hyaluronan	Square (5 × 5 mm)	300	64	25	16
dMNA-BP	Hyaluronan	Square (5 × 5 mm)	300	64	25	16

**Table 2:** Microneedle tip diameters of different MNAs measured using the SEM images. Data are shown as mean ± SD, *n* = 5 (except Tyndall, dMNAs and dMNAs-BP with *n* = 4).

	Bosch	MLT-200	MLT-475	Tyndall	dMNA	dMNA-BP
Tip diameter (µm)	1.2 ± 0.3	7.5 ± 1.0	7.6 ± 0.9	3.8 ± 0.4	4.3 ± 0.7	4.0 ± 0.3

**Table 3:** MNA application parameters used to investigate skin piercing of the six different MNAs via impact insertion.

Pulse Width (8 Bit Value)	Impact Velocity (cm/s)	Impact Energy (mJ)
90	27.40	0.94
104	42.50	2.16
136	70.60	6.06
174	97.40	11.85
221	125.70	19.43
250	138.10	23.84

**Table 4:** Relative standard deviation (RSD) of impact velocity or pressing force calculated for each microneedle array (MNA) application at 125–138 cm/s and 17–25 N.

Reproducibility of Skin Piercing	Bosch (300 µm)	MLT-475 (475 µm)	MLT-200 (200 µm)	Tyndall (300 µm)	dMNA (300 µm)	dMNA-BP (300 µm)
RSD (%) (125–138 cm/s)	1	6	25	2	4	2
RSD (%) (17–25 N)	9	42	121	9	16	7

**Table 5:** EC50 values calculated from the penetration efficiency as a function of impact energy or pressing force. The EC50 values are reported per microneedle array or normalized per microneedle and per microneedle array surface area.

Microneedle Array	Impact Energy Per:			Pressing Force Per:		
	MNA (mJ)	Individual MN (mJ)	mm^2^ (mJ)	MNA (N)	Individual MN (mN)	mm^2^ (mN)
Bosch (300 µm)	0.97	0.002	0.04	1.4	2.4	56.0
MLT-475 (475 µm)	5.47	0.052	0.08	6.3	59.8	91.4
MLT-200 (200 µm)	21.85	0.053	0.31	>25.0 *	>60.1 *	>360.2 *
Tyndall (300 µm)	<1.00 *	<0.063	<0.04	<1.0 *	<62.5 *	<40.0 *
dMNA (300 µm)	1.28	0.080	0.05	<1.0 *	<62.5 *	<40.0 *
dMNA-BP (300 µm)	<1.00 *	<0.063	<0.04	<1.0 *	<62.5 *	<40.0 *

*: the EC50 values could not be calculated because they were higher or lower respectively than the maximum or minimum impact velocity or pressing force used.

## References

[1] Hauri A.M., Armstrong G.L., Hutin Y.J.F. (2004). The global burden of disease attributable to contaminated injections given in health care settings. Int. J. STD AIDS.

[2] Hegde N.R., Kaveri S.V., Bayry J. (2011). Recent advances in the administration of vaccines for infectious diseases: Microneedles as painless delivery devices for mass vaccination. Drug Discov. Today.

[3] Ita K. (2015). Transdermal delivery of drugs with microneedles: Strategies and outcomes. J. Drug Deliv. Sci. Technol..

[4] Kersten G., Hirschberg H. (2007). Needle-free vaccine delivery. Expert Opin. Drug Deliv..

[5] Kim Y.C., Park J.H., Prausnitz M.R. (2012). Microneedles for drug and vaccine delivery. Adv. Drug Deliv. Rev..

[6] Leone M., Monkare J., Bouwstra J.A., Kersten G. (2017). Dissolving Microneedle Patches for Dermal Vaccination. Pharm. Res..

[7] Singh T.R., Dunne N.J., Cunningham E., Donnelly R.F. (2011). Review of patents on microneedle applicators. Recent Pat. Drug Deliv. Formul..

[8] Prausnitz M.R., Mitragotri S., Langer R. (2004). Current status and future potential of transdermal drug delivery. Nat. Rev. Drug Discov..

[9] Arora A., Prausnitz M.R., Mitragotri S. (2008). Micro-scale devices for transdermal drug delivery. Int. J. Pharm..

[10] Engelke L., Winter G., Hook S., Engert J. (2015). Recent insights into cutaneous immunization: How to vaccinate via the skin. Vaccine.

[11] Kalia Y.N., Naik A., Garrison J., Guy R.H. (2004). Iontophoretic drug delivery. Adv. Drug Deliv. Rev..

[12] Prausnitz M.R., Bose V.G., Langer R., Weaver J.C. (1993). Electroporation of mammalian skin: A mechanism to enhance transdermal drug delivery. Proc. Natl. Acad. Sci. USA.

[13] Haq M.I., Smith E., John D.N., Kalavala M., Edwards C., Anstey A., Morrissey A., Birchall J.C. (2009). Clinical administration of microneedles: Skin puncture, pain and sensation. Biomed. Microdevices.

[14] Roxhed N., Samel B., Nordquist L., Griss P., Stemme G. (2008). Painless drug delivery through microneedle-based transdermal patches featuring active infusion. IEEE Trans. Biomed. Eng..

[15] Larraneta E., Lutton R.E.M., Woolfson A.D., Donnelly R.F. (2016). Microneedle arrays as transdermal and intradermal drug delivery systems: Materials science, manufacture and commercial development. Mater. Sci. Eng. R.

[16] Larraneta E., McCrudden M.T.C., Courtenay A.J., Donnelly R.F. (2016). Microneedles: A New Frontier in Nanomedicine Delivery. Pharm. Res..

[17] Tuan-Mahmood T.M., McCrudden M.T., Torrisi B.M., McAlister E., Garland M.J., Singh T.R., Donnelly R.F. (2013). Microneedles for intradermal and transdermal drug delivery. Eur. J. Pharm. Sci..

[18] Van der Maaden K., Luttge R., Vos P.J., Bouwstra J., Kersten G., Ploemen I. (2015). Microneedle-based drug and vaccine delivery via nanoporous microneedle arrays. Drug Deliv. Transl. Res..

[19] Edens C., Dybdahl-Sissoko N.C., Weldon W.C., Oberste M.S., Prausnitz M.R. (2015). Inactivated polio vaccination using a microneedle patch is immunogenic in the rhesus macaque. Vaccine.

[20] Qiu Y., Guo L., Zhang S., Xu B., Gao Y., Hu Y., Hou J., Bai B., Shen H., Mao P. (2015). DNA-based vaccination against hepatitis B virus using dissolving microneedle arrays adjuvanted by cationic liposomes and CpG ODN. Drug Deliv..

[21] Vassilieva E.V., Kalluri H., McAllister D., Taherbhai M.T., Esser E.S., Pewin W.P., Pulit-Penaloza J.A., Prausnitz M.R., Compans R.W., Skountzou I. (2015). Improved immunogenicity of individual influenza vaccine components delivered with a novel dissolving microneedle patch stable at room temperature. Drug Deliv. Transl. Res..

[22] Vrdoljak A., Allen E.A., Ferrara F., Temperton N.J., Crean A.M., Moore A.C. (2016). Induction of broad immunity by thermostabilised vaccines incorporated in dissolvable microneedles using novel fabrication methods. J. Control. Release.

[23] Norman J.J., Arya J.M., McClain M.A., Frew P.M., Meltzer M.I., Prausnitz M.R. (2014). Microneedle patches: Usability and acceptability for self-vaccination against influenza. Vaccine.

[24] Bonificio A., Ghartey-Tagoe E., Gallorini S., Baudner B., Chen G.H., Singh P., O′Hagan D.T., Kommareddy S. (2015). Fabrication of cell culture-derived influenza vaccine dissolvable microstructures and evaluation of immunogenicity in guinea pigs. Vaccine.

[25] Raphael A.P., Prow T.W., Crichton M.L., Chen X.F., Fernando G.I.P., Kendall M.A.F. (2010). Targeted, Needle-Free Vaccinations in Skin using Multi layered, Densely-Packed Dissolving Microprojection Arrays. Small.

[26] Van der Maaden K., Sekerdag E., Jiskoot W., Bouwstra J. (2014). Impact-insertion applicator improves reliability of skin penetration by solid microneedle arrays. AAPS J..

[27] Leone M., Priester M.I., Romeijn S., Nejadnik M.R., Monkare J., O′Mahony C., Jiskoot W., Kersten G., Bouwstra J. (2018). Hyaluronan-based dissolving microneedles with high antigen content for intradermal vaccination: Formulation, physicochemical characterization and immunogenicity assessment. Eur. J. Pharm. Biopharm..

[28] Van der Maaden K., Heuts J., Camps M., Pontier M., Terwisscha van Scheltinga A., Jiskoot W., Ossendorp F., Bouwstra J. (2018). Hollow microneedle-mediated micro-injections of a liposomal HPV E743-63 synthetic long peptide vaccine for efficient induction of cytotoxic and T-helper responses. J. Control. Release.

[29] Verbaan F.J., Bal S.M., van den Berg D.J., Groenink W.H., Verpoorten H., Luttge R., Bouwstra J.A. (2007). Assembled microneedle arrays enhance the transport of compounds varying over a large range of molecular weight across human dermatomed skin. J. Control. Release.

[30] Verbaan F.J., Bal S.M., van den Berg D.J., Dijksman J.A., van Hecke M., Verpoorten H., van den Berg A., Luttge R., Bouwstra J.A. (2008). Improved piercing of microneedle arrays in dermatomed human skin by an impact insertion method. J. Control. Release.

[31] Lee J.W., Park J.H., Prausnitz M.R. (2008). Dissolving microneedles for transdermal drug delivery. Biomaterials.

[32] Van der Maaden K., Jiskoot W., Bouwstra J. (2012). Microneedle technologies for (trans)dermal drug and vaccine delivery. J. Control. Release.

